# An approximate measurement invariance approach to within-couple relationship quality

**DOI:** 10.3389/fpsyg.2014.00983

**Published:** 2014-09-19

**Authors:** Carlo Chiorri, Thomas Day, Lars-Erik Malmberg

**Affiliations:** ^1^Department of Educational Sciences, University of GenoaGenoa, Italy; ^2^Psyche-Dendron AssociationItaly; ^3^Department of Education, University of OxfordOxford, UK

**Keywords:** measurement invariance, Bayesian structural equation modeling, dyadic data, relationship quality, marital satisfaction

## Abstract

This study aimed at demonstrating the usefulness and flexibility of the Bayesian structural equation modeling approximate measurement invariance (BSEM-AMI) approach to within-couple data. The substantive aim of the study was investigating partner differences in the perception of relationship quality (RQ) in a sample of intact couples (*n* = 435) drawn from the first sweep of the Millenium Cohort Study. Configural, weak and strong invariance models were tested using both maximum likelihood (ML) and BSEM approaches. As evidence of a lack of strong invariance was found, full and partial AMI models were specified, allowing nine different prior variances or “wiggle rooms.” Although we could find an adequately fitting BSEM-AMI model allowing for approximate invariance of all the intercepts, the two-step approach proposed by Muthén and Asparouhov (2013b) for identifying problematic parameters and applying AMI only to them provided less biased results. Findings similar to the ML partial invariance model, led us to conclude that women reported a higher RQ than men. The results of this study highlight the need to inspect parameterization indeterminacy (or alignment) and support the efficacy of the two-step approach to BSEM-AMI.

## Introduction

In this study we present a worked example of the usefulness and flexibility of the recently developed Bayesian structural equation modeling approximate measurement invariance analysis (BSEM-AMI, Muthén and Asparouhov, 2013b) in addressing a common issue in relationship research, i.e., testing mean differences in partners' perception of relationship quality (RQ). This is a special case of gender differences testing, since the data from each individual are not unrelated to the data from every other individual in the study, as partners are nested within couples. This violates the assumption of independent errors and implies that, as we discuss in the Analytic strategy section, the unit of analysis has to be the couple, with women and men being different (but identifiable) raters of the same relationship. While our aim is not to draw definite conclusions about the long debated issue of partner differences in the perception of RQ, we offer to relationship researchers an example of a principled analytical approach to address it. For our didactic purposes we used partners' scores on a 7-item version of the Golombok-Rust Inventory of Marital State (Rust et al., 1986, 1990), which is included in the first sweep of University of London, Institute of Education, Centre for Longitudinal Studies (2012). As the psychometric properties of this short version have not been comprehensively tested, this study also provides evidence of its reliability, unidimensionality and partial measurement invariance across partners in intact couples.

### The substantive focus

Relationship quality (RQ), also referred to as marital quality, marital satisfaction or dissatisfaction, marital characterization, marital discord, marital conflict or relationship satisfaction is a key measure in family and developmental research. It has been linked to personal outcomes such as psychological and physical health of the partners, and with some crucial family outcomes such as domestic violence, poor parenting, and poor adjustment of children (Grych and Fincham, 2001; Fincham, 2003). Partners who lead a happy relationship are healthier (but see Robles et al., 2014), tend to communicate well with each other, are good-enough parents who raise their children authoritatively, and run less risk of marital breakdown (for more information see Section Introduction of the Supplementary Materials). Bradbury et al. (2000) highlighted the crucial role that RQ plays in sustaining individual and family-level well-being. A society benefits from such strong marital bonds that are formed and maintained as they provide a robust basis for bringing up children. Healthy children, parents and communities provide the rationale behind the need to “develop empirically defensible interventions for couples that prevent marital distress and divorce” (p. 964).

It is therefore important to understand with some precision how we can adequately measure the quality of relationships within couples. The use of reliable measures could assist practitioners (e.g., family therapists) to draw fine-tuned differences in partners' perceptions of their couple-life. In family research there are models in which the dependencies between partners are modeled, such as the Actor-Partner-Interdependence-Model (APIM; Kenny, 1996; Kenny and Cook, 1999), as well as models in which within-couple agreement (correlations) or discrepancies (mean-level differences) are specified (e.g., Luo et al., 2008).

RQ has been operationally defined as a global evaluation of the relationship along several dimensions, including self-reported satisfaction with the relationship, attitudes toward one's partner, and levels of hostile and negative behavior (Robles et al., 2014). Numerous measures have been developed to assess it, from single-item measures (e.g., “How happy is your relationship with your partner, all things considered?” rated on a 7-point Likert-type scale, included in the National Child Development Study in UK), to multi-item measures, such as the Locke-Wallace Marital Adjustment Test (MAT, Locke and Wallace, 1959) or the Dyadic Adjustment Scale (DAS, Spanier, 1976, revised by Busby and Christensen, 1995, for use with distress and non-distressed couples) (for reviews see Child Trends, 2003; Bronte-Tinkew et al., 2004; Reynolds et al., 2014). In this study we focused on the Golombok-Rust Inventory of Marital State (GRIMS, Rust et al., 1986, 1990), whose 7-item shortened version was included in the first sweep (2003) of the Millenium Cohort Study (MCS). Rust et al. (1986) developed the GRIMS for use in couple counseling centers as a measure of change before and after treatment and was initially intended as a companion to the Golombok-Rust Inventory of Sexual Satisfaction (GRISS, Rust and Golombok, 1985). The original 28-item GRIMS allows to measure relationship change over time and to highlight relationship difficulties and focuses on two domains of the relationship, (1) shared interests, communication, sex, warmth, roles and decision making, and coping, and (2) beliefs about and attitudes toward relationships, behavior in the relationship and agreement with the partner. The 7-item GRIMS was developed to meet the need for a shorter measure of RQ to be included in the MCS questionnaire while retaining the content validity of the original version. Using archival data, the items to be included in the final version were chosen in order to (a) retain the framework of the original blueprint as much as possible; (b) obtain a similar number of positive and negative items and (c) achieve adequate corrected item-total correlations. The shortened scale was tested on a standardization sample of 266 individuals, and results showed good internal consistency (Cronbach's α = 0.86 in both women and men), and no significant skewness or kurtosis (Rust, personal communication, 2014^1^). As for its criterion validity, previous findings from the MCS in relation to outcomes of RQ revealed that, women more satisfied with their relationship use less harsh discipline, parents in happier relationships spent more time with their children, women happier in relationships had children with higher British Ability Scale naming vocabulary scores and low RQ is linked to more behavioral problems (Jones, 2010).

One issue that has been extensively investigated in relation to RQ is whether there are gender differences, i.e., whether partners systematically experience different levels of RQ. As reported by Jackson et al. (2014) in their recent meta-analytic study, since Bernard (1972)'s seminal work it has long been assumed that women experience significantly less relationship satisfaction than men, but despite a number of studies supported this assumption, evidence for a lack of difference has also been provided (see Jackson et al., 2014 for a review). Jackson et al. (2014) concluded that there was a high average correlation of RQ scores between husband and wife pairs (0.51), and that wives were 51% less likely to be satisfied than their husbands only in couples undergoing marital therapy, whereas the difference was not significantly different from zero in community-based couples, especially in intact couples. This meta-analysis did not include studies that used the GRIMS. Rust et al. (1986, 1990) reported mixed results about gender differences in the 28-item scale scores: in the 1986 study, men obtained higher scores than women in the pilot sample and in clinical samples, while in the 1990 study men's scores were lower in clinical samples and equal in a sample of attendees at a general practitioners clinic. In the development study of the 7-item GRIMS, Rust (personal communication, 2014^1^) did not find significant gender differences in raw scores.

### The methodological focus

Both Jackson et al. (2014) and Rust et al. (1986, 1990) studies tested gender differences in RQ observed scores under the untested assumption that all the RQ measures included in the analysis showed measurement invariance across partners, i.e., the underlying measurement model of RQ measures was equivalent for both women and men. In particular, a crucial assumption in the comparison of RQ scores across spouses is, beyond the invariance of factor loadings, the invariance of item intercepts, i.e., whether the mean differences based on the latent construct are reflected in each of the individual items used to infer it. In other words, if the level of partner differences in RQ varies substantially from item to item for different items used to infer the construct, then the partner differences based on the corresponding latent construct should be considered idiosyncratic to the particular items used to infer RQ (i.e., differential item functioning). If this turns out to be true, results would suggest that conclusions about differences in RQ do not generalize over the set of items used in the instrument and the interpretation of latent mean comparisons among partners would be compromised (van de Schoot et al., 2012). In other words, even if partners rate the same items about the same relationship, their scores cannot be compared because the instrument does not measure the same construct in the same way.

Despite the wide use of RQ measures in surveys and research, very few studies have compared their factor structures across partners. One exception is South et al. (2009)'s study that demonstrated support for factorial invariance of the DAS across spouses. As pointed out by the authors, having established invariance of the DAS across gender, it can be concluded that any differences between men and women (as they found for dyadic consensus and affectional expression, with men scoring lower than women) can be interpreted as arising from actual differences in relationship adjustment, not that the instrument is measuring different constructs in the two groups. Besides, being able to reliably establish that there are systematic differences in scores between women and men would imply that different norms might be needed to interpret scores from either spouse. To the best of our knowledge no study has addressed this issue about the 7-item GRIMS, nor whether there are gender differences in scores. To this end, the aim of this study was to test its measurement invariance and investigate whether there are gender differences in its scores in a sample of intact couples from the first sweep of the MCS.

One frequent issue about measurement invariance is what to do when, after finding support for the ability of the a priori model to fit the data in each group without invariance constraints (*configural invariance*) and for the invariance of the factor loadings (*weak* or *metric invariance*), the model that imposes equality on item intercepts (*strong* or *scalar invariance*), does not fit, thus preventing a meaningful test of latent score differences. Muthén and Christoffersson (1981) suggested that it is possible to test invariance when only some of the parameters are invariant, and they termed this “partial” measurement invariance. Byrne et al. (1989) argued that full invariance is not necessary for performing further invariance tests and substantive analyses and proposed that mean comparisons would be meaningful if weak and strong invariance have been satisfied for at least two items per latent trait. Actually, the estimates of trait mean differences will be more accurately estimated with imposed partial invariance constraints, since the trait mean estimates are adjusted for the fact that only partial, not full, invariance characterizes the data: in other words, allowing the intercepts to vary automatically excludes the non-invariant items from the estimation of latent means (Cheung and Rensvold, 2000). Another approach to the problem, named Approximate Measurement Invariance (AMI), has been recently described by Muthén and Asparouhov (2012a, 2013b) and successfully implemented by van de Schoot et al. (2013). This method uses Bayesian structural equation models (BSEM) in which *exact* zero constraints can be replaced with *approximate* zero constraints based on substantive theories. In other words, differences in item intercepts that in confirmatory factor analysis would be constrained to be zero, under AMI can be estimated with some so-called “wiggle room” (Muthén and Asparouhov, 2012b), implying that very small differences are allowed and thus finding a compromise between zero and no constraints, through which both model fit and latent mean comparison can be established. A Bayesian approach involves the use of (1) prior distributions, which represent background knowledge about the parameters of a model, (2) the likelihood function of the data containing the information about the parameters from the data, and (3) a posterior distribution, which contains one's updated knowledge balancing prior knowledge with observed data.

If most of the items show small differences in intercepts, the application of full AMI is recommended, with “small” implying that parameters of substantive interest do not change in a meaningful way if MI does not fully hold (van de Schoot et al., 2013). In most applications, however, the number of non-invariant parameters might be small with respect to the number of actually invariant ones, but this does not prevent an unacceptable model fit. In these cases Muthén and Asparouhov (2013b) and van de Schoot et al. (2013) recommended a two-step procedure in which parameters that are different between groups (and hence are the major sources of misfit) are detected in step 1, for example by using modification indices provided by the ML estimation, and are allowed to be non-invariant to the extent imposed by partial AMI in step 2. A technical description of the statistical features of these models is beyond the scope of this paper (see Muthén and Asparouhov, 2013b; van de Schoot et al., 2013, for a gentle introduction), which is instead to provide a didactic example of how to establish strong measurement invariance using AMI in the particular case of within-couple data.

## Materials and methods

### Sample

The sample for this study was UK-based and drawn from the first sweep of the Millennium Cohort Study (MCS). The MCS is a longitudinal study drawing its sample from all live births in the United Kingdom over 12 months, in England, Wales, Scotland and Northern Ireland. The first sweep took place in 2003 when the children were aged 9 months, and later follow-ups at the ages of 3, 5, 7, and 11 (University of London, Institute of Education, Centre for Longitudinal Studies, 2012; for details, see Plewis, 2007). In this study we included families which were present at Sweep 1 (*n* = 18.552). If families had twin or triplet births the child coded as cohort member “a” was included in our sample. As we were interested in ratings of both partners within couples, we selected two-parent-figure families, in which both parent figures were present, were of opposite sex, were one generation older than the child, were not blood relatives, and were biological parents (for details, see Malmberg and Flouri, 2011). For the purpose of our demonstration of the AMI procedure we used a sub-sample of parents from the Northern Ireland-advantaged stratum (*n* = 527). Although the procedure presented here can handle missing data either with a full information or a multiple imputation approach, the implications of dealing with missingness were beyond the scope of the paper. As we aimed to provide a relatively straightforward example of the application of BSEM-AMI, we screened for missing data on the GRIMS and used a listwise sample of 435 couples.

### Measure

The 7-item GRIMS provides a self-reported assessment of the quality of a couple's relationship asking participants to rate seven items [(1) My partner is sensitive to and aware of my needs; (2) My partner doesn't listen to me anymore; (3) I'm sometimes lonely when I'm with my partner; (4) Our relationship is full of joy and excitement; (5) I wish there was more warmth and affection between us; (6) I suspect we are on the brink of separation; (7) We can make up quickly after an argument] on a 5-point, Likert-type agreement scale (1 = Strongly Agree, 5 = Strongly Disagree). Scores of items 1, 4, and 7 were reverse scored before performing the analyses so that higher item scores corresponded to higher RQ.

### Analytic strategy

**Figure 1:**
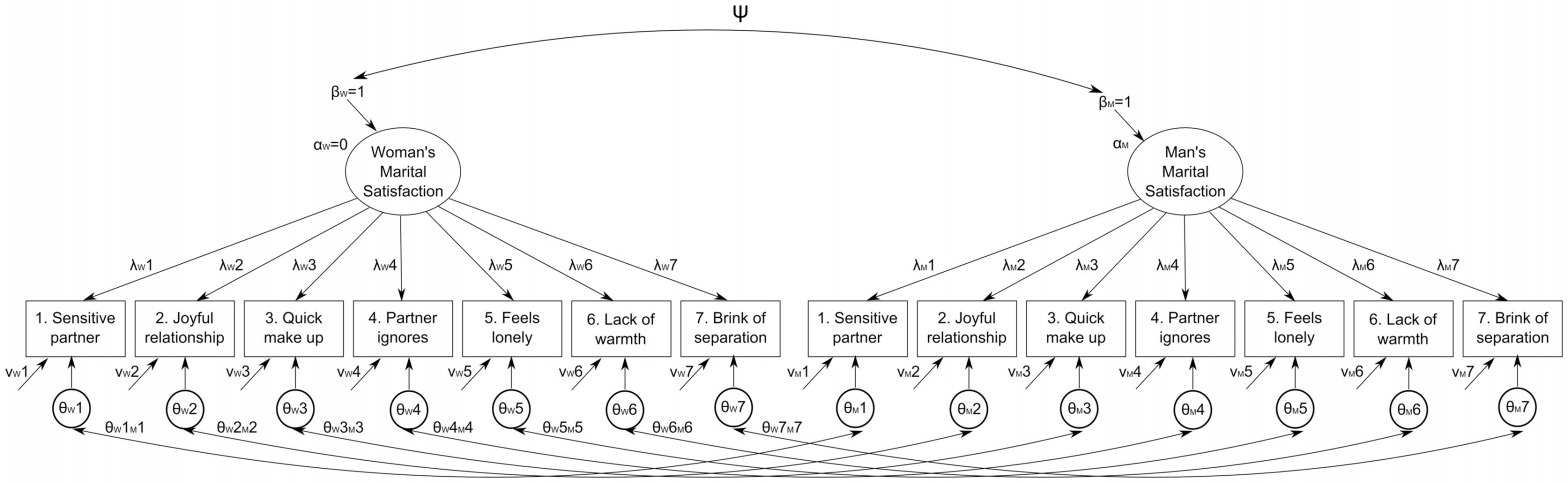
**Baseline model used for the confirmatory factor analytic invariance analysis between women's (W subscript) and men's (M subscript) ratings of marital satisfaction**. Note that Mplus notation is used, i.e., α, factor mean; β, factor variance; ψ, factor correlation; λ, factor loading; ν, item intercept; θ, item uniqueness.

Before testing measurement invariance across partners, we tested the fit of the hypothesized one-factor model for the GRIMS separately for men and women through the “classical” confirmatory factor analysis (CFA) using maximum likelihood estimation with robust standard errors (MLR) to address the relatively non-normal distributions of item scores (Table S1 in Supplementary Information). The fit of the models was evaluated considering the root-mean-square error of approximation (RMSEA), the Tucker–Lewis index (TLI), and the comparative fit index (CFI), as operationalized in Mplus v7 (Muthén and Muthén, 1998-2012) in association with the MLR estimator. For both the TLI and CFI, values greater than 0.90 and 0.95, respectively, typically reflect acceptable and excellent fit to the data. For the RMSEA, values less than 0.05 and 0.08 reflect a close fit and a reasonable fit to the data, respectively (Marsh et al., 2004). After finding that the expected measurement model fitted adequately in both partners, we specified the sequence of invariance models as in the Meredith (1993) tradition. However, as pointed out by South et al. (2009), testing measurement invariance on paired groups of observations as couple data is different from testing it on independent groups, since both partners are reporting on the same relationship. Instead of testing the same, e.g., one-factor measurement model on two different groups defined by a grouping variable, we therefore modeled the data at the couple level, i.e., the unit of analysis was the couple, and partners were treated as different raters on the same relationship. This means that, for each couple, both women's and men's ratings were on the same line of data. This model is basically equivalent to a single-group two-correlated-factor model in which the items of the scale are considered twice, as indicators of women's and men's marital satisfaction (Figure 1).

This also implies that the systematic residual variance (uniqueness) in each pair of identical items between parents is expected to covary because of the identical nature of the item pair (Brown, 2006, chap. 7, e.g., the residual variance in the item “My partner doesn't listen to me any more” for women should covary with the same item for men). Hence, the model with correlated uniqueness (*θ*_*WiMi*_s in Figure 1) should have resulted in a substantial improvement in fit over the model without the correlated uniqueness (e.g., Burns et al., 2008). The fit of invariance models was evaluated with the same criteria stated above, while model comparisons were performed using the Satorra-Bentler Scaled Chi-Square Difference Test (Satorra and Bentler, 2001) but, considering that this test suffers the same problems (i.e., sample size dependency) as the chi-square test used to test goodness of fit that led to the development of fit indices, we also considered as support for the more parsimonious model a change in CFI of less than 0.01 or a change in RMSEA of less than 0.015 (Chen, 2007). In case of rejection of the more parsimonious (i.e., constrained) model we inspected modification indices in Mplus output to find the least invariant parameter and re-specified the model letting it to be non-invariant. This procedure was iterated until no more parameters were suggested to be non-invariant.

For Bayesian models we used default prior settings, i.e., normal prior distributions for the intercepts and factor loadings with a prior mean of zero and a prior variance of 10^10^, and an inverse gamma distribution for the (residual) variance terms with hyperparameters −1 and zero; note that this model is similar to the configural invariance model because it implies practically no “real” prior constraint, and the following Mplus Analysis settings: BCONVERGENCE = 0.01; BITERATIONS = 1,000,000 (20,000); PROCESSOR = 2; CHAINS = 2; BSEED = 167. As indices of model fit we used the posterior predictive *p*-value (PPP) and the 95% confidence interval (CI) for the difference in the *f* statistic for the real and replicated data (see Muthén and Asparouhov, 2012a). An acceptably fitting model should have shown a PPP higher than 0.05 and a 95% CI of the replicated chi-square values that included zero. Nine different wiggle rooms σ^2^ (0.50, 0.25, 0.125, 0.05, 0.025, 0.01, 0.005, 0.001, 0.0005) were specified for non-invariant parameters, with smaller values allowing smaller wiggle rooms and therefore a closer approximation of the “classical” invariance model. Given the didactic aim of this paper, we initially tested full AMI models, in which all parameters of interest were allowed to be non-invariant to the extent allowed by the wiggle room, and then turned to partial AMI, following the two-step procedure recommended by Muthén and Asparouhov (2013b) and described above.

The data and all syntax files are available as supplementary materials.

## Results

If we simply compared women's and men's observed scores on the GRIMS with a paired-sample *t*-test, we would have concluded that women (*M* = 29.23, *SD* = 3.63, Cronbach's α = 0.73) tend to be systematically more satisfied than men (*M* = 28.19, *SD* = 3.67, α = 0.73) [*t*_(434)_ = 5.08, *p* < 0.001, *d* = 0.29] and that the two scores are only moderately correlated (*r* = 0.31, *p* < 0.001). However, as stated above, this result is meaningful only if strong measurement invariance holds. The one-factor measurement model for the GRIMS had an acceptable fit for women [SBχ^2^(14) = 33.258, *p* = 0.002, Scaling Correction Factor [SCF] = 1.071, CFI = 0.944, TLI = 0.916, RMSEA = 0.056) and optimal for men [SBχ^2^(14) = 16.862, *p* = 0.264, SCF = 1.491, CFI = 0.990, TLI = 0.985, RMSEA = 0.022]. Factor score determinacies, i.e., validity coefficients computed as the correlation between factor score estimates and their respective latent factors, were 0.868 and 0.871, respectively, suggesting a high (>0.80, Gorsuch, 1983) degree of convergence of observed scores on the scale and the latent individuals' scores. Raykov (1997)'s composite reliabilities were in both cases 0.740.

As a first step for testing measurement invariance we compared the configural invariance models with and without correlated uniquenesses. As shown in Table 1, the fit of both models was acceptable, suggesting an adequate ability of the a priori one-factor measurement model to fit the data in each partner without invariance constraints. However, the model with the correlated uniqueness fitted statistically and substantially better than the one without, and was thus chosen as the baseline model for the invariance tests.

**Table 1:** **Goodness of fit of maximum likelihood with robust standard errors (MLR) full and partial invariance models**.

**Invariance model**	**SBχ^2^**	***df***	***p***	**SCF**	**RMSEA**	**CFI**	**TLI**	***cd***	**TRd**	**Δ*df***	***p***	**Mean difference estimate**				**Simulation**					**Bias**	
												**α**	***SE***	***p***	***d***	**AVG**	***SD***	***SE avg***	**95% Cover**	**% Sig**.	***M bias (%)***	***SE Bias (%)***
Configural no CUs	97.948	76	0.046	1.112	0.026	0.973	0.967															
Configural with CUs	82.052	69	0.135	1.111	0.021	0.984	0.979	1.112	15.889	7	0.026											
Weak with CUs	90.371	76	0.125	1.115	0.021	0.982	0.979	1.154	8.319	7	0.305											
Strong with CUs	127.238	82	0.001	1.108	0.036	0.944	0.937	1.017	39.534	6	<0.001	−0.341	0.070	<0.001	0.25	−0.344	0.072	0.068	0.938	0.998	0.79	−5.58
Partial1^∧^ with CUs	108.190	81	0.024	1.110	0.028	0.966	0.962	0.946	22.081	1	<0.001											
Partial2^*^ with CUs	94.677	80	0.126	1.110	0.021	0.982	0.979	1.110	13.513	1	<0.001	−0.502	0.075	<0.001	0.35	−0.505	0.079	0.075	0.945	1.000	0.56	−4.20

SBχ^2^, Satorra-Bentler scaled chi-square; df, degrees of freedom; SCF, Scaling Correction Factor; RMSEA, Root Mean Square Error of Approximation; CFI, Comparative Fit Index; TLI, Tucker-Lewis Index; cd, difference test scaling correction; TRd, Satorra-Bentler scaled chi-square difference test; Δdf, degrees of freedom difference; CU, correlated uniquenesses;

∧ Intercept of item 4 was not invariant;

* *Intercepts of items 3 and 4 were not invariant. SB scaled chi-square difference tests are referred to the model in the above line*.

We then constrained the loadings for identical items to be equal between parents (*weak* or *metric invariance*). If identical items have statistically equivalent loadings, then the identical items show the same amount of increase between parents for the same amount of increase on the latent factor. As shown in Table 1, this constraint did not significantly affected model fit, hence we concluded that weak invariance held. However, the comparison of latent means is appropriate only if it can be shown that also intercepts of the same items are invariant between partners, i.e., *strong* or *scalar invariance* holds. When factor loadings and intercepts are invariant, at any point along the factor continuum the same level of the factor results in statistically equivalent average scores on identical items between parents, namely, any observed score differences between parents on identical items is not due to partner bias but rather to actual differences on the factor mean. Due to model identification issues it is not possible to estimate the two latent means simultaneously, hence we fixed at zero the women's mean and estimated the men's mean, which thus represented the mean difference of men's RQ scores with respect to women's. Table 1 shows that constraining to invariance all item intercepts led to a substantial and significant decrease of fit, although this remained acceptable. We concluded that full strong invariance could not be assumed, thus undermining the possibility of reliably comparing latent means.

The inspection of modification indices from the strong invariance model indicated that intercept of item 4 should be allowed to vary across parents. The fit of the model allowing for the partial invariance of this intercept (Partial1 in Table 1) was higher than the fit of the strong invariance model, but it was still lower than that of the weak invariance model [SBΔχ^2^(5) = 18.692, *p* = 0.002 and ΔCFI > 0.01]. Following the modification indices, in a subsequent model (Partial2) we permitted the intercept of item 3 be invariant, too. This model fitted significantly and substantially better than Partial1 model and its fit did not differ from the fit of the weak invariance model [SBΔχ^2^(5) = 4.264, *p* = 0.371 and ΔCFI < 0.01]. The standardized estimated latent mean difference was −0.502, and was statistically different from zero, suggesting that women were more satisfied with their relationship than men, although with a small (*d* < 0.50) effect size.

We then turned to Bayesian SEM, and re-analyzed the configural, weak and strong invariance models (FMI1–FMI3 in Table 2).

**Table 2:** **Goodness of fit, estimated latent mean differences and their estimated bias for Bayesian full and partial invariance models**.

**Model**	**95% CI χ^2^**	**PPP**	**Median absolute intercept difference (range)**	**Mean difference estimate**				**Simulation**					**Bias**	
				**α**	***SE***	***p***	***d***	**AVG**	***SD***	***SE* Avg**	**95% Cover**	**% Sig**.	***M* Bias (%)**	***SE* Bias (%)**
**FULL INVARIANCE**														
FMI1 configural	−16.183 58.471	0.106		–	–	–		–	–		–	–	–	
FMI2 weak	−12.794 57.036	0.111	0.131 (0.010–0.409)	–	–	–		–	–		–	–	–	
FMI3 strong	20.836 93.107	0.001	0.000 (0.000–0.000)	−0.340	0.066	<0.001	0.27	−0.348	3.161	0.593	0.314	0.682	2.24	−81.23
AFMI1 σ^2^ = 0.5	−14.605 59.226	0.119	0.088 (0.014–0.224)	−0.279	0.533	0.284	0.03	−0.340	0.360	0.425	0.952	0.123	22.01	18.01
AFMI2 σ^2^ = 0.25	−14.219 58.635	0.119	0.071 (0.007–0.196)	−0.315	0.377	0.194	0.04	−0.344	0.194	0.346	0.995	0.080	9.24	78.25
AFMI3 σ^2^ = 0.125	−14.811 59.127	0.122	0.062 (0.001–0.184)	−0.333	0.280	0.123	0.06	−0.340	0.117	0.266	1.000	0.119	1.98	127.76
AFMI4 σ^2^ = 0.05	−15.240 61.016	0.107	0.051 (0.003–0.183)	−0.354	0.185	0.027	0.10	−0.342	0.077	0.186	1.000	0.410	−3.50	142.00
AFMI5 σ^2^ = 0.025	−15.163 61.056	0.108	0.048 (0.004–0.178)	−0.355	0.138	0.005	0.13	−0.342	0.069	0.142	1.000	0.827	−3.80	104.18
AFMI6 σ^2^ = 0.01	−14.855 61.681	0.106	0.044 (0.003–0.158)	−0.350	0.101	<0.001	0.18	−0.340	0.066	0.104	0.997	0.976	−2.94	56.97
AFMI7 σ^2^ = 0.005	−13.530 63.776	0.086	0.036 (0.001–0.134)	−0.346	0.084	<0.001	0.21	−0.338	0.065	0.087	0.987	0.995	−2.43	34.41
AFMI8 σ^2^ = 0.001	−0.366 77.232	0.028	0.016 (0.000–0.062)	−0.341	0.069	<0.001	0.26	−0.333	0.064	0.071	0.967	0.999	−2.38	9.97
AFMI9 σ^2^ = 0.0005	6.400 83.511	0.012	0.009 (0.000–0.038)	−0.341	0.067	<0.001	0.26	−0.333	0.064	0.068	0.961	1.000	−2.29	6.88
PARTIAL INVARIANCE^*^														
PMI strong	−13.704 62.416	0.095	−0.214 −0.264	−0.499	0.071	<0.001	0.36	−0.494	0.070	0.075	0.967	1.000	−1.10	6.28
APMI1 σ^2^ = 0.5	−11.802 61.498	0.090	−0.213 −0.263	−0.499	0.071	<0.001	0.36	−0.492	0.071	0.074	0.967	1.000	−1.34	5.09
APMI2 σ^2^ = 0.25	−11.718 61.594	0.090	−0.212 −0.262	−0.497	0.074	<0.001	0.35	−0.489	0.071	0.074	0.965	1.000	−1.59	5.25
APMI3 σ^2^ = 0.125	−11.799 61.592	0.090	−0.209 −0.259	−0.495	0.074	<0.001	0.35	−0.485	0.070	0.074	0.964	1.000	−2.12	5.12
APMI4 σ^2^ = 0.05	−12.175 61.725	0.087	−0.199 −0.250	−0.489	0.074	<0.001	0.34	−0.472	0.069	0.073	0.959	1.000	−3.46	6.24
APMI5 σ^2^ = 0.025	−11.671 62.672	0.087	−0.186 −0.236	−0.479	0.074	<0.001	0.33	−0.454	0.068	0.072	0.954	1.000	−5.14	6.33
APMI6 σ^2^ = 0.01	−9.953 64.009	0.081	−0.155 −0.203	−0.457	0.072	<0.001	0.33	−0.419	0.066	0.071	0.934	1.000	−8.29	6.49
APMI7 σ^2^ = 0.005	−7.646 67.449	0.062	−0.122 −0.165	−0.433	0.071	<0.001	0.32	−0.388	0.065	0.069	0.921	1.000	−10.39	6.45
APMI8 σ^2^ = 0.001	6.166 81.574	0.017	−0.045 −0.067	−0.374	0.069	<0.001	0.28	−0.340	0.064	0.067	0.933	0.999	−9.14	4.35
APMI9 σ^2^ = 0.0005	12.325 87.639	0.008	−0.025 −0.039	−0.358	0.068	<0.001	0.27	−0.334	0.064	0.067	0.945	1.000	−6.70	4.07

95% CI χ^2^, 95% confidence interval for the difference between the observed and the replicated χ^2^; PPP, posterior predictive p-value; α, mean latent score difference parameter; SE, standard error of α; p, significance of α; d, effect size; AVG, average of estimated mean latent score differences over the replications; SD, standard deviation of mean difference parameter estimate over the replications; SE avg, average of the estimated standard errors for the mean difference parameter estimate over the replications; 95% cover, proportion of replications for which the 95% CI included the hypothesized population value α; % sig., proportion of datasets for which the 95% CI did not include zero, i.e., the percentage of datasets for which it can be concluded that AVG is larger than zero in the population; M bias, (AVG-α)/α^*^100; SE bias, (SE avg-SD)/SD; FMI, Full measurement invariance; AFMI, Approximate full measurement invariance; PMI, partial measurement invariance; APMI, approximate partial measurement invariance; σ^2^, prior variance of intercepts for all pairs of items;

* *Intercepts of items 3 and 4 were not invariant, values in the intercept difference column are the differences between women's–men's intercepts for items 3 and 4, respectively*.

As shown in Table 2, the “classical” strong invariance model (all intercepts constrained to equality across partners, FMI3), did not fit the data, since the posterior predictive *p*-value was <0.05, and the 95% CI of the replicated chi-square values did not include zero, whereas the configural (FMI1) and weak (FMI2) invariance model adequately fitted the data, but did not allow to compare the latent means. We thus resorted to full AMI and we restricted intercept differences by specifying the 9 prior distributions described above (same “wiggle room” for all intercepts, AFMI1–AFMI9 in Table 2). Results are shown in the upper part of Table 2, and values in the median absolute intercept difference column show that restricting the wiggle room led to smaller intercept differences. Models with prior variance 0.001 (AFMI8) and 0.0005 (AFMI8) should be rejected, since either their 95% CI for the difference between the observed and the replicated χ ^2^ did not include zero or their ppp-value was lower than 0.05, or both. However, it is interesting to note that, among the acceptably fitting models, the estimate of the factor mean difference was not always significantly different from zero, probably due to alignment (see Discussion). It became so only when σ^2^ was 0.05 (AFMI4) or lower, with small effect sizes.

The Mplus output for Bayesian AMI models provides the equivalent of modification indices in ML MI models, i.e., the DIFFERENCE OUTPUT, in which the deviations from the mean and their significance for non-invariant parameters are shown. Deviations from the mean were significant for item 4 in model AMI5, for items 4 and 5 in model AMI6, for items 3, 4, and 5 in model AMI7 and for item 4 in model AMI8 (Table S2 in Supplementary Materials). In order to compare the results with the ML partial invariance models, we tested approximate partial measurement invariance (APMI) models allowing a wiggle room only for items 3 and 4 (PMI1 and APMI1–APMI9 in Table 2), while constraining to equality the intercepts of the other items. Note that instead of the modification index approach of relaxing one equality restriction at a time, we followed Muthén and Asparouhov (2013b)'s suggestion to relax all misfitting equalities, since when they are not too many they do not have much effect on the point estimates nor on the identification of the model, although they might slightly increase the standard errors. As it is shown in the bottom part of Table 2, an adequate fit was obtained when σ^2^ ranged from 0.50 to 0.005 (APMI1–APMI6), and, in these cases, all estimates of factor mean differences were statistically significant. Effect sizes were larger than in full invariance models and similar to the effect size of the partial invariance ML model, but still in the small range.

Given the mixed pattern of results about the estimate of latent mean differences, we wondered which result should be trusted. Hence, we investigated the possible bias in the comparison of latent means through a Monte Carlo simulation study. van de Schoot et al. (2013) investigated the possible bias in the comparison of latent means as a result of applying the approximate MI model by performing a simulation study in which seven populations with different sets of (assumed) true values were specified. Since we could not know the true population values, we decided to use the estimates obtained in testing the model as population values to explore the stability of the models and the appropriate convergence of parameter estimates to the assumed population parameters. Results were obtained with ESTIMATOR = ML and with ESTIMATOR = BAYES. For the latter we used PROCESSORS = 2; BCONVERGENCE = 0.01; BITERATIONS = (5000); BSEED = 167; and the default priors for both full and partial invariance models.

For each population we generated 1000 datasets. We considered an estimate as acceptably unbiased if (1) the empirical standard deviation of the 1000 estimated mean differences was lower than 0.10; (2), the relative mean bias of the estimate defined as (AVG-α)/α)^*^100, where AVG is average mean obtained from the simulation study and α is the assumed population value, did not exceed 10% (e.g., van de Schoot et al., 2013); (3) the standard error bias for the parameter for the mean difference parameter did not exceed 5% (Muthén and Muthén, 2002); (4) 95% coverage, i.e., the proportion of replications for which the 95% CI included the population value, was at least 95%; (5), the significance criterion, i.e., the proportion of datasets for which the 95% CI of the factor mean difference estimate did not include zero and was therefore statistically significant, was close to 1.00. Results are shown in the rightmost columns of Table 2.

Among the adequately fitting full invariance models, no full invariance model met the criteria stated above, since the standard error bias was always higher than 5%. The simulation of the full strong invariance model with ML did not meet criteria 3 and 4 (Table 1). On the other hand, PMI and APMI models 1–5 appeared to provide sufficiently unbiased results, although the standard error bias was slightly over the cut-off (Table 2). The simulation of the partial strong invariance model with ML basically met all criteria, with only criterion 4 borderline met (Table 1).

Taken together, these results suggest that there are gender differences in the perception of RQ as measured by the 7-item GRIMS in intact couples, with women reporting higher scores.

It is interesting to note that estimates and significance of the factor correlation were stable at slightly less than 0.42 throughout all models, suggesting that a higher relationship satisfaction in women tends to be associated with a higher relationship satisfaction in men, and that all the criteria stated above for assessing bias were basically satisfied (only criterion 4 showed borderline values; see Table S3 in the Supplementary Materials).

## Discussion

The aim of this study was to demonstrate the usefulness and flexibility of the BSEM-AMI for investigating measurement invariance, particularly addressing the issue of possible lack of strong invariance in within-couple data. Specifically, we provided an example of how a common issue in relationship research, i.e., partner differences in the perception of relationship quality, can be addressed with this methodology. We applied BSEM-AMI to ratings on the 7-item GRIMS in a sample of intact couples drawn from the MCS database, and the results suggested that women perceived a higher RQ than men (although with a small effect size), somewhat contradicting the results of a recent meta-analysis (Jackson et al., 2014) that showed that in intact couples there are no substantial differences in RQ. However, this meta-analysis did not include any study using the 7-item GRIMS, nor could we screen our sample for couples in marital therapy, for which partner differences in RQ are known to exist, though in the other direction (Jackson et al., 2014). As a limitation to the generalizability of the results for the substantive issue of this paper, it must also be considered that the data used in this study were collected in a specific subgroup of couples, i.e., Northern-Irish, advantaged, intact couples 9 months after the birth of a child. This sample is similar to South et al. (2009)'s study, in which data from intact couples with long-term marriages included in the Minnesota Twin Family Study were used and results similar to ours were found, as women reported higher levels of dyadic adjustment. These results are also consistent with those of Shapiro et al. (2000), who reported that marital satisfaction was significantly higher for women who became mothers than for men who became fathers. However, since other studies suggested that marital satisfaction is lower among the individuals who are most responsible for the child, which in most cases is the mother (e.g., Hochschild, 1989), and in light of the aforementioned limitations, we cannot consider our results conclusive as to the general question of whether women and men differ in the perception of RQ.

In pursuing the substantive focus of this investigation, we showed how BSEM-AMI can be successfully applied to address it. As we found lack of support for a strong invariance model, i.e., a model that assumes that all item intercepts, along with factor loadings, are perfectly invariant across partners and allows a valid comparison of latent scores, through BSEM-AMI we could release the assumption of a *zero* difference between intercepts and allow a “wiggle-room” for it, i.e., an *approximately* zero difference. AMI models that allowed this wiggle room for all intercepts showed an acceptable fit and suggested that differences in GRIMS scores could exist between partners. However, as shown in Table 2, the significance of the mean difference parameter α increased as the wiggle room got smaller. This result might be due to alignment, i.e., a parameterization indeterminacy (Muthén and Asparouhov, 2013b). In other words, the BSEM-AMI tries to find a solution in which the variance across partners for a measurement parameter is small. Since the wiggle room is prior variance distribution, and thus allows a pre-determined range of variation for parameter estimates, the method is more effective when there is a large degree of minor non-invariance and parameters deviations from invariance are in opposite directions and can largely cancel each other out (Muthén and Asparouhov, 2013b). In specifying full AMI models, we assumed that the wiggle room was the same for all intercepts: in these cases, however, if there is an item with a relatively large difference whereas the difference is relatively small in all the others, the BSEM small-variance prior for the parameter differences tends to pull the deviating parameter toward the average of the parameters for both partners. This means that the deviating parameter will be smaller and the invariant parameters larger than their true values. With intercepts misestimated, the factor means and factor variances are misestimated too (Muthén and Asparouhov, 2013b). Our simulation studies suggested that this might have been the case of the present investigation (due to the relatively large difference in intercepts for items 3 and 4), given the BSEM analysis we used is not expected to always recover parameter values used to simulate the data. Although the mean difference estimates showed a negligible bias, the standard error bias was large. Muthén and Asparouhov (2013a) noted that this does not necessarily mean that the model does not fit the data, but that an equally well-fitting solution with a possibly simpler interpretation due to another simplicity criterion may be available. They also suggest to detect non-invariant items and relax equalities only on them, since this will lead to the recovering of the parameter values. When we resorted to partial invariance models, and we identified in intercepts of items 3 and 4 the major sources of lack of fit, results were much more stable and unbiased, although it seemed that a very small wiggle room still lead to inadequately fitting models (see AMPI models 8–9 in Table 2). Actually, the ML partial invariance model also provided a good fit and unbiased estimated of mean difference and its standard error, suggesting that, in this case, a more “classical” approach would have led to similar conclusions as the “new” Bayesian approach.

The results of this study seem to support the efficacy of the two-step approach suggested by Muthén and Asparouhov (2013b) and van de Schoot et al. (2013), in which parameters that are different between groups are detected in step 1 through modification indices and are allowed to be non- (or approximately) invariant in step 2. However, it should be noted that partial measurement invariance has the main shortcoming that the parameters to be freed are identified with an ex-post facto procedure. This might raise the issue of capitalization on chance when the sample is small and/or not representative of the population, and undermine the generalizability of the results, especially in those studies in which the measurement model of a scale is investigated. Muthén and Asparouhov (2012a) pointed out that while ML modification indices inform about model improvement when a single parameter is freed and can lead to a long series of modifications, BSEM can inform about model modification when all parameters are freed and does so in a single step: their simulations showed sufficient power to detect model misspecification in terms of 95% Bayesian credibility intervals not covering zero. Nontheless, they also warn that, as with ML model modification, BSEM model modification should be supported by substantive interpretability. However, in most research contexts (e.g., developing a new questionnaire) one cannot know in advance the sources of non-invariance and whether deviations from invariance will eventually bias the substantive conclusions or not: hence we recommend to carefully check the stability of model estimates through simulation studies.

Another issue that it is worth noting, although not a focus of this study, is that the indices of model fit for Bayesian and ML SEM do not always overlap. The reason for which we chose the Northern Ireland-advantaged subsample of the whole MCS dataset is that it was the only one in which the fit indices of the one-factor measurement model for the GRIMS were adequate for both estimation methods. When we considered the largest stratum of the MCS, i.e., the England advantaged stratum (*n* = 3830), the configural invariance model fitted adequately with ML, but it was not even remotely adequate with BSEM (see Table S4 in Supplementary Materials). As an example, we drew random smaller subsamples and retested model fit, finding no substantial changes in ML indices, but a gradual approach to acceptable values for BSEM. Muthén and Asparouhov (2012a) warn that the posterior PPP does not behave like a *p*-value for a chi-square test of model fit (e.g., Hjort et al., 2006), hence the Type I error is not 5% for a correct model. Since there is not a theory for how low the PPP can be before the model is significantly inadequately fitting at a certain level, Muthén and Asparouhov (2012a) consider it more akin to an SEM fit index rather than a chi-square test. Using simulations they found that PPP performed better than the ML likelihood-ratio chi-square test at small sample sizes where ML typically inflates chi-square and that it was less sensitive than ML to ignorable deviations from the correct model thus concluding that PPP seems to have sufficient power to detect important model misspecifications, that might go unnoticed when using ML.

### Conflict of interest statement

The authors declare that the research was conducted in the absence of any commercial or financial relationships that could be construed as a potential conflict of interest.

## Footnotes

^1^Rust, J. (2014). *Personal Communication*, July 14, 2014.
