# Supplementary material for: An approximate measurement invariance approach to within-couple relationship quality
Source: Front Psychol. 2014 Sep 19;5:983. doi: 10.3389/fpsyg.2014.00983 (PMC4168678; doi:10.3389/fpsyg.2014.00983)
Supplement: Supplementary file 1 [file DataSheet1.DOC]

***Supplementary Material***

**An approximate measurement invariance approach to within-couple relationship quality**

**Carlo Chiorri^1^, Thomas Day^2^, and Lars-Erik Malmberg^2^**

**^1^University of Genova, Department of Educational Sciences, Genova, Italy,**

**^2^University of Oxford, Department to Education, Oxford, UK;**

*** Correspondence:** Carlo Chiorri, University of Genova, Department of Educational Sciences, Corso A. Podestà, 2, 16128 Genova, Italy, carlo.chiorri@unige.it

**1. Supplementary text**

**Relationship quality: antecedents and outcomes**

Many studies have sought to explore antecedents of relationship quality (RQ). It has been found that individuals with a history of depression during adolescence are more likely to experience lower levels of RQ (Gotlib et al, 1998): more specifically, diagnosed depression during adolescence significantly predicted subsequent diminished marital satisfaction and, for male participants, elevations in marital disagreements. Additionally, it has been found that personality traits can predict RQ: for example, a woman's relationship happiness is predicted by her partner's low negative emotionality, high positive emotionality, and high constraint, whereas a man's relationship happiness is predicted only by his partner's low negative emotionality. Besides, each partner's personality contributes independently to relationship outcomes but not in a synergistic way (Robins et al. 2000). Also higher levels of the trait mindfulness predict higher relationship satisfaction (Barnes et al, 2007) and positive links have been found between communication and satisfaction (DeMaris and Leslie, 1984). It has also been reported that the transition to parenthood results in a modest, but significant unfavourable changes in RQ (Belsky et al, 1983) whereas other studies have highlighted that some difficulties or adverse life events can result in increased relationship quality: for example, Gritz et al, (1990) found that the experience of testicular cancer for the male partner strengthened RQ for many couples. Economic factors have also been found to have a significant impact on RQ, such that job characteristics can predict marital quality (Hughes et al., 1992) and economic pressure predicts emotional distress for both husbands and wives: specifically, these emotional distresses are positive predictors of conflict in the relationship and increased levels of martial distress (Cogner et al., 1999).

The implications of RQ for children's outcomes have additionally been extensively explored. RQ during infancy has been found to predict the frequency of behavioural problems of children (Benzies et al, 2004) and has been linked with a more child-father attachment, especially in girls, whilst the security of the child-mother attachment was not significantly related to the degree of RQ (Goldberg and Easterbrook, 1984). DeVito and Hopkins (2001) reported a negative correlation between permissive parenting and RQ and found links between low levels of RQ and the likelihood of children to display disruptive behaviour. Further, it has been found that parents with high RQ display higher levels of parenting satisfaction (Rogers and White, 1998). Recently, a study exploring brain activity responses to facial images of individuals' spouse found links between RQ and both psychological and physical health, finding a positive association between RQ and activation in several neural regions in the brain related to reward, motivation, stress control and effective regulation, whilst being negatively correlated with areas related to severe depression (Acevedo et al, 2012).

**References**

Acevedo, B. P., Aron, A., Fisher, H. E., and Brown, L. L. (2012). Neural correlates of marital satisfaction and well-being: Reward, empathy and affect. *Clin. Neuropsychiatry* 9, 20-31. Available online at: http://www.helenfisher.com/downloads/articles/Acevedo%20et%20al-MarSatisfaction.pdf.

Barnes, S., Warren Brown, K., Krusemark, E., and Campbell, W. K. (2007). The role of mindfulness in romantic relationship satisfaction and responses to relationship stress. *J. Marital Fam. Ther*. 33, 482-500. doi: 10.1111/j.1752-0606.2007.00033.x.

Belsky, J., Spanier, G. B., and Rovine, M. (1983). Stability and change in marriage across the transition to parenthood. *J. Marriage Fam* 45, 567-577. doi: 10.2307/351661

Benzies, K. M., Harrison, M. J., and Magill-Evans, J. (2004). Parenting stress, marital quality, and child behavior problems at age 7 years. *Public Health Nurs.* 21, 111-121. doi: 10.1111/j.0737-1209.2004.021204.x.

Cogner, R. D., Rueter, M. A., and Elder, G. H. (1999). Couple resilience to economic pressure. *J. Pers. Soc. Psychol.* 76, 54-71. doi: 10.1037/0022-3514.76.1.54.

DeMaris, A., and Leslie, G. R. (1984). Cohabitation with the future spouse: Its influence upon marital satisfaction and communication. *J. Marriage Fam.* 46, 77-84. doi: 10.2307/351866.

Devito, C., and Hopkins, J. (2001). Attachment, parenting, and marital dissatisfaction as predictors of disruptive behavior in preschoolers. *Dev. Psychopathol.* 13, 215-231. doi: 10.1017/S0954579401002024.

Goldberg, W. A. and Easterbrooks, M. A. (1984). Role of marital quality in toddler development. *Dev. Psychol.* 20*,* 504-514. doi: 10.1037/0012-1649.20.3.504.

Gotlib, I. H., Lewinsohn, P. M., and Seeley, J. R. (1998). Consequences of depression during adolescence: Marital status and marital functioning in early adult-hood*. J. Abnorm. Psychol.* 107, 686-690. doi: 10.1037/0021-843X.107.4.686.

Gritz, E. R., Wellisch, D. K., Siau, J., and Wang, H. (1990). Long-term effects of testicular cancer on marital relationships. *Psychosomatics* 31*,* 301-312. doi: 10.1016/S0033-3182(90)72168-8.

Hughes, D., Galinsky, E., and Morris, A. (1992). The effects of job characteristics on marital quality: Specifying liking mechanisms. *J. Marriage Fam.* 54*,* 31-42. doi: 10.2307/353273.

Robins, R. W., Caspi, A. and Moffitt, T. E. (2000). Two personalities, one relationship: Both partners' Pers. traits shape the quality of their relationship. *J. Pers. Soc. Psychol.* 79*,* 251-259. doi: 10.1037/0022-3514.79.2.251.

Rogers, S. J., and White, L.K. (1998). Satisfaction with parenting: The role of marital happiness, fam. structure, and parent's gender. *J. Marriage Fam.* 60, 293-308. doi: 10.2307/353849.

**2. Supplementary Tables**

**Table S1 Descriptive statistics for the 7-item** Golombok Rust Inventory of Marital State

| Item | Min | Max | M | SD | SK | KU |
| --- | --- | --- | --- | --- | --- | --- |
| Women |  |  |  |  |  |  |
| 1. My partner is sensitive to and aware of my needs | 1 | 5 | 4.19 | 0.79 | -1.04 | 1.45 |
| 1. My partner doesn’t listen to me any more (r) | 1 | 5 | 4.18 | 0.94 | -1.34 | 1.66 |
| 1. I’m sometimes lonely when I’m with my partner (r) | 1 | 5 | 4.18 | 0.89 | -1.04 | 0.57 |
| 1. Our relationship is full of joy and excitement | 1 | 5 | 3.72 | 0.88 | -0.53 | 0.13 |
| 1. I wish there was more warmth and affection between us. (r) | 1 | 5 | 3.92 | 0.99 | -0.90 | 0.32 |
| 1. I suspect we are on the brink of separation. (r) | 1 | 5 | 4.81 | 0.55 | -4.16 | 22.31 |
| 1. We can make up quickly after an argument | 1 | 5 | 4.23 | 0.80 | -1.60 | 4.23 |
| Total score | 16 | 35 | 29.23 | 3.63 | -0.60 | 0.24 |
|  |  |  |  |  |  |  |
| Men |  |  |  |  |  |  |
| 1. My partner is sensitive to and aware of my needs | 1 | 5 | 4.01 | 0.75 | -0.87 | 1.51 |
| 1. My partner doesn’t listen to me any more (r) | 1 | 5 | 3.97 | 0.88 | -1.13 | 1.69 |
| 1. I’m sometimes lonely when I’m with my partner (r) | 1 | 5 | 4.14 | 0.87 | -1.20 | 1.55 |
| 1. Our relationship is full of joy and excitement | 1 | 5 | 3.73 | 0.80 | -0.47 | 0.44 |
| 1. I wish there was more warmth and affection between us (r) | 1 | 5 | 3.51 | 1.07 | -0.39 | -0.74 |
| 1. I suspect we are on the brink of separation (r) | 1 | 5 | 4.73 | 0.66 | -3.59 | 15.68 |
| 1. We can make up quickly after an argument | 1 | 5 | 4.10 | 0.88 | -1.39 | 2.52 |
| Total score | 14 | 35 | 28.19 | 3.67 | -0.46 | 0.65 |

Note: *n*=435; Min=minimum; Max=maximum; M=mean; SD=standard deviation; SK=skewness; KU=kurtosis; (r)=reverse item (statistics refer to reversed scores)

**Table S2 Difference output for approximate measurement invariance models in Table 2**

|  | AMI5 | | | |  | AMI6 | | | |  | AMI7 | | | |
| --- | --- | --- | --- | --- | --- | --- | --- | --- | --- | --- | --- | --- | --- | --- |
| Item | $\bar{\nu}$ | SD | ΔW | ΔW |  | $\bar{\nu}$ | SD | ΔW | ΔW |  | $\bar{\nu}$ | SD | ΔW | ΔW |
| 1 | 4.180 | 0.042 | 0.010 | -0.010 |  | 4.179 | 0.036 | 0.011 | -0.011 |  | 4.179 | 0.034 | 0.009 | -0.009 |
| 2 | 4.156 | 0.046 | 0.024 | -0.024 |  | 4.155 | 0.041 | 0.022 | -0.022 |  | 4.154 | 0.039 | 0.018 | -0.018 |
| 3 | 4.249 | 0.047 | -0.064 | 0.064 |  | 4.247 | 0.041 | -0.055 | 0.055 |  | 4.247 | 0.038 | -0.046* | 0.046* |
| 4 | 3.813 | 0.047 | -0.088* | 0.088* |  | 3.812 | 0.040 | -0.078* | 0.078* |  | 3.812 | 0.037 | -0.067* | 0.067* |
| 5 | 3.839 | 0.061 | 0.078 | -0.078 |  | 3.839 | 0.052 | 0.071* | -0.071* |  | 3.839 | 0.049 | 0.059* | -0.059* |
| 6 | 4.808 | 0.027 | -0.002 | 0.002 |  | 4.808 | 0.024 | -0.001 | 0.001 |  | 4.807 | 0.024 | -0.001 | 0.001 |
| 7 | 4.221 | 0.036 | 0.012 | -0.012 |  | 4.220 | 0.033 | 0.011 | -0.011 |  | 4.220 | 0.032 | 0.009 | -0.009 |

Note: $\bar{\nu}$=mean intercept; SD=standard deviation; ΔW and ΔM: deviations from the mean for women and men

**Table S3** Goodness of fit, estimated factor correlations and their estimated bias for Bayesian full and partial invariance models

| Model | 95% CI χ^2^ | PPP | median absolute intercept difference (range) | Factor correlation estimate | | |  | Simulation | | | | |  | Bias | |
| --- | --- | --- | --- | --- | --- | --- | --- | --- | --- | --- | --- | --- | --- | --- | --- |
|  |  |  |  | *ψ* | SE | p |  | AVG | SD | SE avg | 95%cover | % sig. |  | M bias | SE bias |
| Full invariance | | | | | | | | | | | | | | | |
| FMI3 Strong | 20.836 93.107 | .001 | 0.000 (0.000-0.000) | .419 | .059 | <.001 |  | .412 | .056 | .057 | .950 | 1.000 |  | -1.01% | 1.78% |
| AFMI1 σ^2^ = 0.5 | -14.605 59.226 | .119 | 0.088 (0.014-0.224) | .416 | .058 | <.001 |  | .412 | .057 | .057 | .947 | 1.000 |  | -0.91% | 1.06% |
| AFMI2 σ^2^ = 0.25 | -14.219 58.635 | .119 | 0.071 (0.007-0.196) | .416 | .058 | <.001 |  | .412 | .057 | .057 | .948 | 1.000 |  | -0.94% | 1.06% |
| AFMI3 σ^2^ = 0.125 | -14.811 59.127 | .122 | 0.062 (0.001-0.184) | .416 | .058 | <.001 |  | .412 | .057 | .057 | .946 | 1.000 |  | -0.91% | 0.88% |
| AFMI4 σ^2^ = 0.05 | -15.240 61.016 | .107 | 0.051 (0.003-0.183) | .416 | .059 | <.001 |  | .412 | .057 | .057 | .948 | 1.000 |  | -0.91% | 1.23% |
| AFMI5 σ^2^ = 0.025 | -15.163 61.056 | .108 | 0.048 (0.004-0.178) | .417 | .059 | <.001 |  | .413 | .057 | .057 | .949 | 1.000 |  | -0.89% | 1.23% |
| AFMI6 σ^2^ = 0.01 | -14.855 61.681 | .106 | 0.044 (0.003-0.158) | .416 | .059 | <.001 |  | .413 | .057 | .057 | .948 | 1.000 |  | -0.84% | 1.06% |
| AFMI7 σ^2^ = 0.005 | -13.530 63.776 | .086 | 0.036 (0.001-0.134) | .416 | .059 | <.001 |  | .413 | .057 | .057 | .946 | 1.000 |  | -0.77% | 1.23% |
| AFMI8 σ^2^ = 0.001 | -0.366 77.232 | .028 | 0.016 (0.000-0.062) | .417 | .059 | <.001 |  | .414 | .057 | .058 | .944 | 1.000 |  | -0.79% | 1.23% |
| AFMI9 σ^2^ = 0.0005 | 6.400 83.511 | .012 | 0.009 (0.000-0.038) | .417 | .059 | <.001 |  | .414 | .057 | .058 | .942 | 1.000 |  | -0.77% | 1.23% |
| Partial invariance | | | | | | | | | | | | | | | |
| PMI1Strong | -13.704 62.416 | .095 | -0.214 -0.264 | .419 | .057 | <.001 |  | .419 | .415 | .057 | .057 | .947 |  | -0.86% | 0.70% |
| APMI1 σ^2^ = 0.5 | -11.802 61.498 | .090 | -0.213 -0.263 | .419 | .057 | <.001 |  | .419 | .416 | .057 | .057 | .949 |  | -0.84% | 1.42% |
| APMI2 σ^2^ = 0.25 | -11.718 61.594 | .090 | -0.212 -0.262 | .419 | .057 | <.001 |  | .419 | .415 | .057 | .057 | .951 |  | -0.86% | 1.42% |
| APMI3 σ^2^ = 0.125 | -11.799 61.592 | .090 | -0.209 -0.259 | .419 | .057 | <.001 |  | .419 | .415 | .057 | .057 | .949 |  | -0.86% | 1.24% |
| APMI4 σ^2^ = 0.05 | -12.175 61.725 | .087 | -0.199 -0.250 | .419 | .057 | <.001 |  | .419 | .415 | .057 | .057 | .945 |  | -0.86% | 1.06% |
| APMI5 σ^2^ = 0.025 | -11.671 62.672 | .087 | -0.186 -0.236 | .419 | .057 | <.001 |  | .419 | .416 | .057 | .057 | .948 |  | -0.84% | 1.24% |
| APMI6 σ^2^ = 0.01 | -9.953 64.009 | .081 | -0.155 -0.203 | .418 | .057 | <.001 |  | .418 | .415 | .057 | .057 | .947 |  | -0.81% | 1.06% |
| APMI7 σ^2^ = 0.005 | -7.646 67.449 | .062 | -0.122 -0.165 | .418 | .057 | <.001 |  | .418 | .415 | .057 | .057 | .946 |  | -0.79% | 0.88% |
| APMI8 σ^2^ = 0.001 | 6.166 81.574 | .017 | -0.045 -0.067 | .417 | .057 | <.001 |  | .417 | .414 | .057 | .058 | .948 |  | -0.84% | 1.23% |
| APMI9 σ^2^ = 0.0005 | 12.325 87.639 | .008 | -0.025 -0.039 | .417 | .057 | <.001 |  | .417 | .413 | .057 | .058 | .949 |  | -0.86% | 1.23% |

Note: 95% CI χ^2^=95% confidence interval for the difference between the observed and the replicated χ^2^; PPP=posterior predictive p-value; *ψ*=factor correlation;SE=standard error of *ψ*; p=significance of *ψ*; AVG=average of estimated factor correlation over the replications; SD=standard deviation of factor correlation estimate over the replications; SE avg=average of the estimated standard errors for the factor correlation estimate over the replications; 95% cover=proportion of replications for which the 95% CI included the hypothesized population value *ψ*; % sig.= proportion of datasets for which the 95% CI did not include zero, i.e., the percentage of datasets for which it can be concluded that AVG is larger than zero in the population; *ψ* bias=(AVG-α)/α*100; SE bias=(SE avg-SD)/SD; FMI=Full measurement invariance; AFMI=Approximate full measurement invariance; PMI=partial measurement invariance; APMI=approximate partial measurement invariance; σ^2^=prior variance of intercepts for all pairs of items; *=intercepts of item 3 and 4 were not invariant, values in the intercept difference column are the differences between women's−men's intercepts for items 3 and 4, respectively.

**Table S4 Goodness of fit of Bayesian and Robust Maximum Likelihood confirmatory factor analysis models for the configural invariance model of the Golombok-Rust Inventory of Marital States in the England-advantaged stratum (n=3830) of the Millenium Cohort Study as a function of the number of cases**

|  | Bayesian | | |  | Robust Maximum Likelihood | | | | | | |
| --- | --- | --- | --- | --- | --- | --- | --- | --- | --- | --- | --- |
| n | 95% CI χ^2^ | | PPP |  | SB-χ^2^ | df | p | SCF | RMSEA | CFI | TLI |
| 3830 | 511.939 | 600.406 | <.001 |  | 576.381 | 69 | <.001 | 1.144 | .044 | .952 | .936 |
| 1917 | 282.830 | 364.992 | <.001 |  | 358.878 | 69 | <.001 | 1.140 | .047 | .944 | .926 |
| 976 | 109.838 | 187.982 | <.001 |  | 199.914 | 69 | <.001 | 1.151 | .044 | .948 | .932 |
| 470 | 19.021 | 98.265 | <.001 |  | 119.710 | 69 | <.001 | 1.083 | .040 | .963 | .952 |
| 263 | 26.637 | 104.229 | <.001 |  | 127.331 | 69 | <.001 | 1.116 | .057 | .924 | .900 |

Note: n=number of cases; 95% CI χ^2^=95% confidence interval for the difference between the observed and the replicated χ^2^; PPP=posterior predictive p-value; SBχ^2^=Satorra-Bentler scaled chi-square; df=degrees of freedom; SCF=Scaling Correction Factor; RMSEA=Root Mean Square Error of Approximation; CFI=Comparative Fit Index; TLI=Tucker-Lewis Index;
